# Supplementary material for: Emergence and spread of the barley net blotch pathogen coincided with crop domestication and cultivation history
Source: PLoS Genet. 2024 Jan 29;20(1):e1010884. doi: 10.1371/journal.pgen.1010884 (PMC10852282; doi:10.1371/journal.pgen.1010884)
Supplement: S1 Fig — Kruskal-Wallis test with post-hoc pairwise Wilcoxon was used to identify significant differences (p < 0.05) between the groups (Table D in S1 Table). (PDF) [file pgen.1010884.s002.pdf]

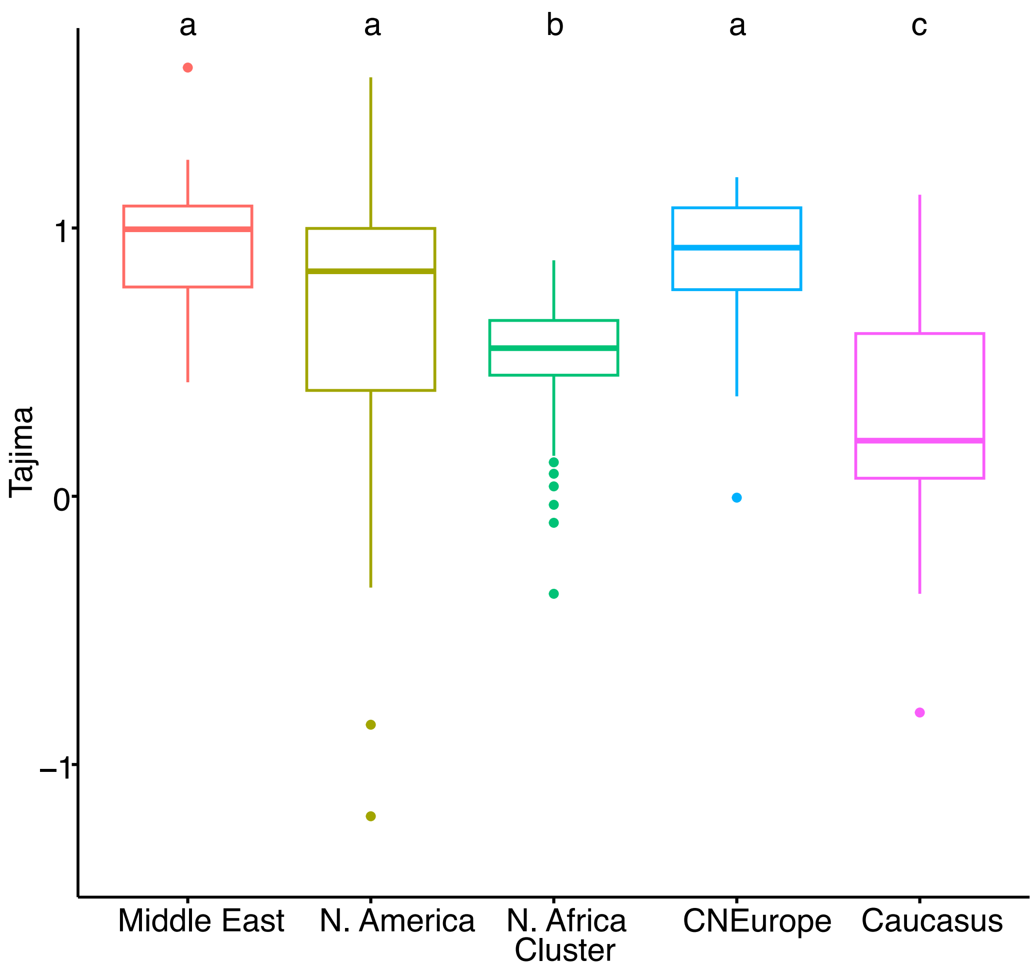

Figure S1: Tajima's D of *P. teres* f. *teres* populations in each geographic region. Pairwise Wilcoxon was used to identify significant differences ($p < 0.05$) between the groups (Table S4).
