# Supplementary material for: Emergence and spread of the barley net blotch pathogen coincided with crop domestication and cultivation history
Source: PLoS Genet. 2024 Jan 29;20(1):e1010884. doi: 10.1371/journal.pgen.1010884 (PMC10852282; doi:10.1371/journal.pgen.1010884)
Supplement: S2 Fig — (PDF) [file pgen.1010884.s003.pdf]

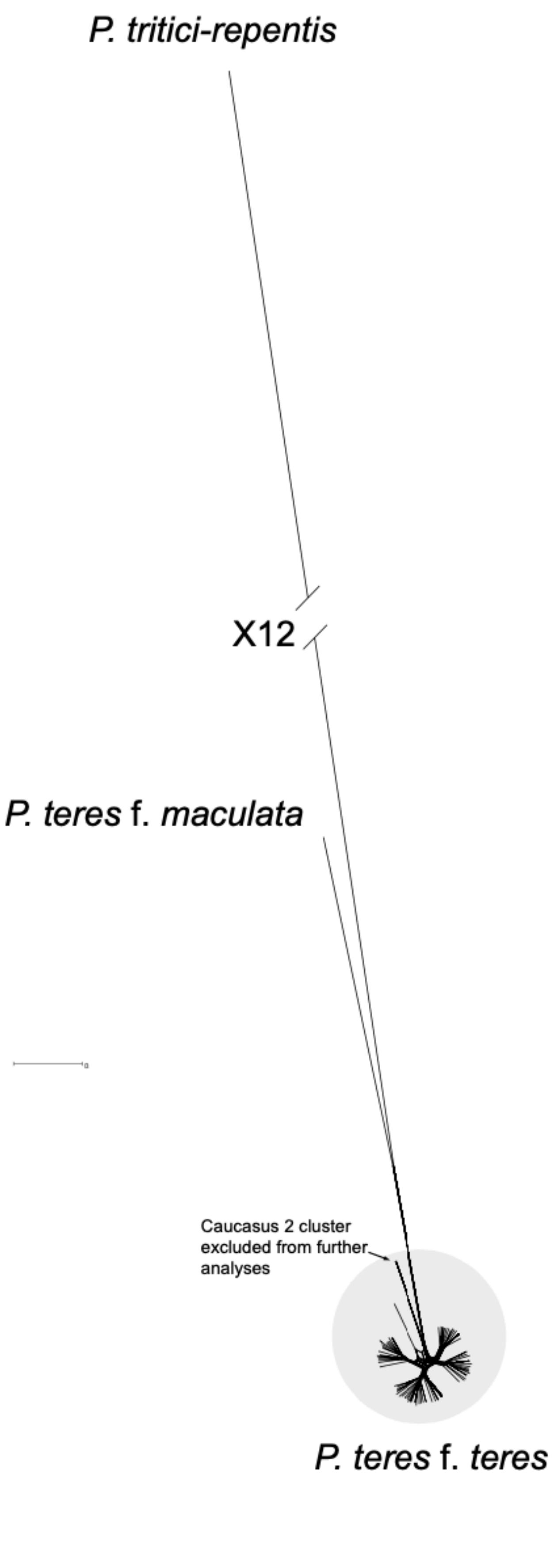

Figure S2: NeighbourNet tree shows the evolutionary relationship between *P.teres* f. *teres, P.teres* f. *maculata* and *P. tritici-repentis.*
