# Supplementary material for: Emergence and spread of the barley net blotch pathogen coincided with crop domestication and cultivation history
Source: PLoS Genet. 2024 Jan 29;20(1):e1010884. doi: 10.1371/journal.pgen.1010884 (PMC10852282; doi:10.1371/journal.pgen.1010884)
Supplement: S3 Fig — (PDF) [file pgen.1010884.s004.pdf]

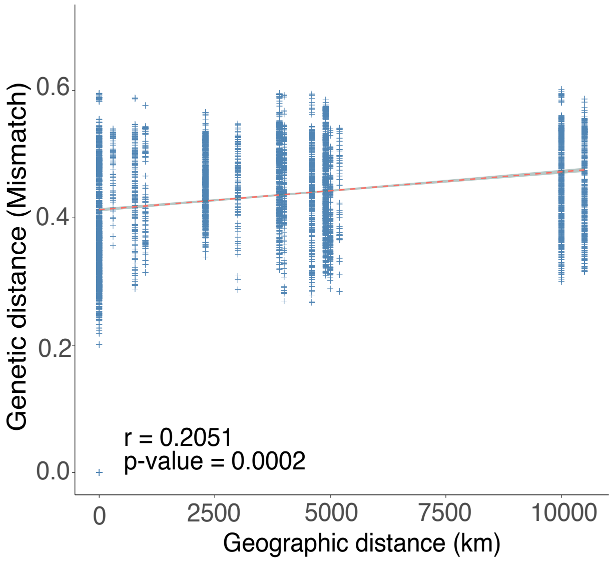

Figure S3: Mantel test shows significant corelation between geographic and genetic distance (Number of permutations: 9,999, Pearson corelation coefficient).
