# Supplementary material for: Emergence and spread of the barley net blotch pathogen coincided with crop domestication and cultivation history
Source: PLoS Genet. 2024 Jan 29;20(1):e1010884. doi: 10.1371/journal.pgen.1010884 (PMC10852282; doi:10.1371/journal.pgen.1010884)
Supplement: S4 Fig — (PDF) [file pgen.1010884.s005.pdf]

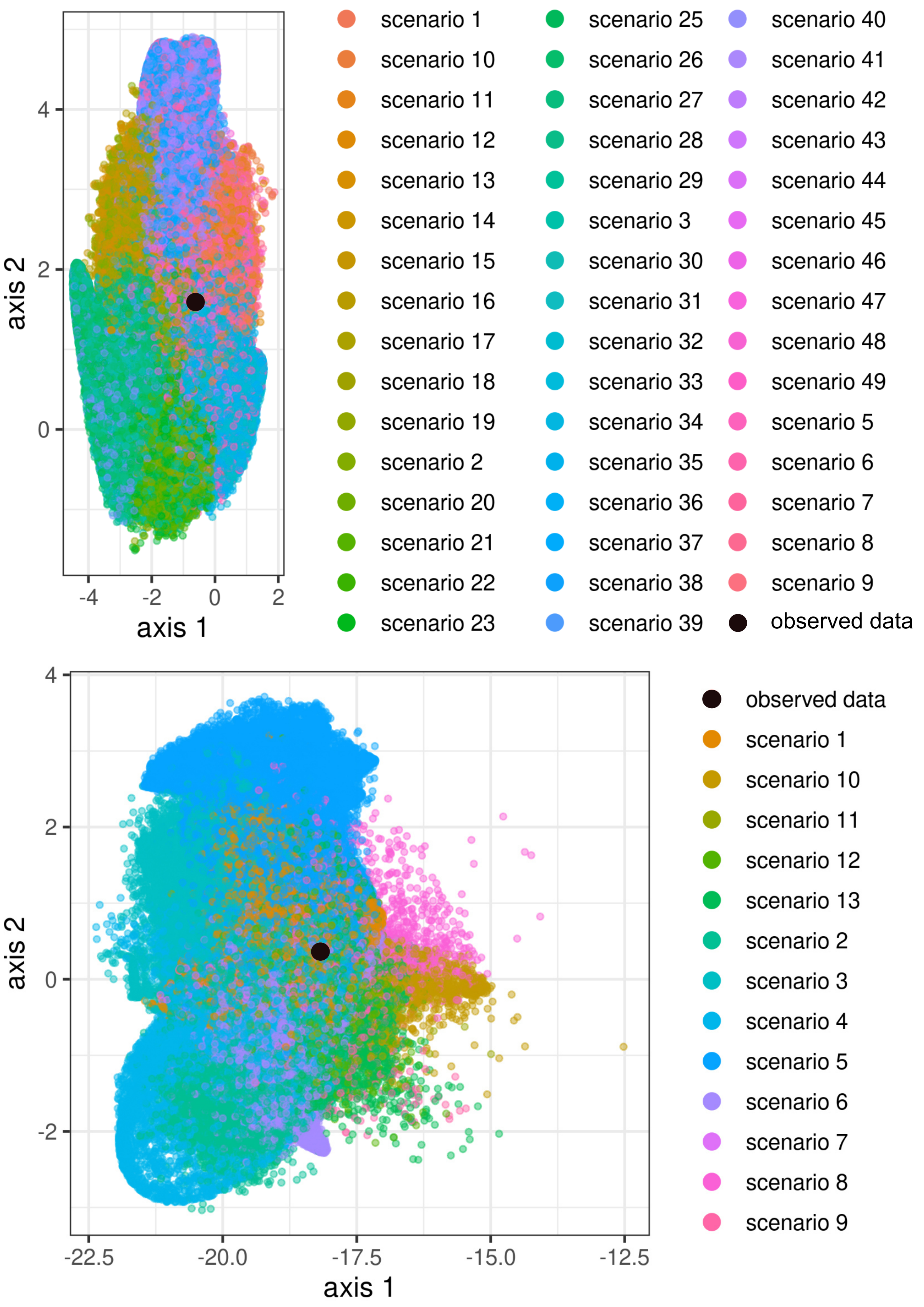


Figure S4: Scenarios of Family 1 tested on the first step of ABC-RF to assess the early divergence and evolutionary relationships of Middle East, Caucasus, North African populations. Pop 1, pop2, and pop 3 correspond to Middle East, N. Africa and Caucasus, respectively.

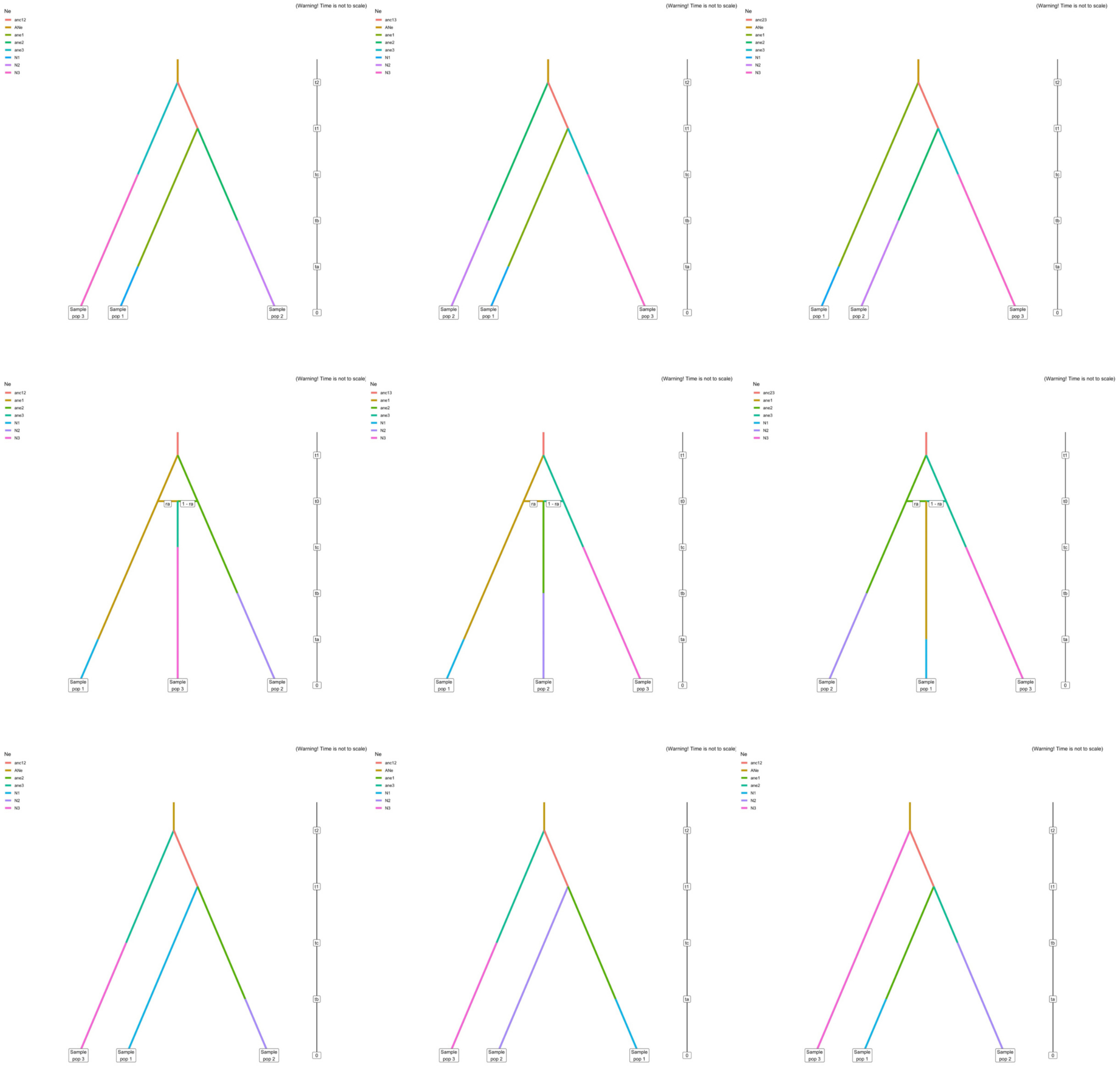


Figure S4 (Continued): Scenarios of Family 1 tested on the first step of ABC-RF to assess the early divergence and evolutionary relationships of Middle East, Caucasus, North African populations. Pop 1, pop2, and pop 3 correspond to Middle East, N. Africa and Caucasus, respectively.

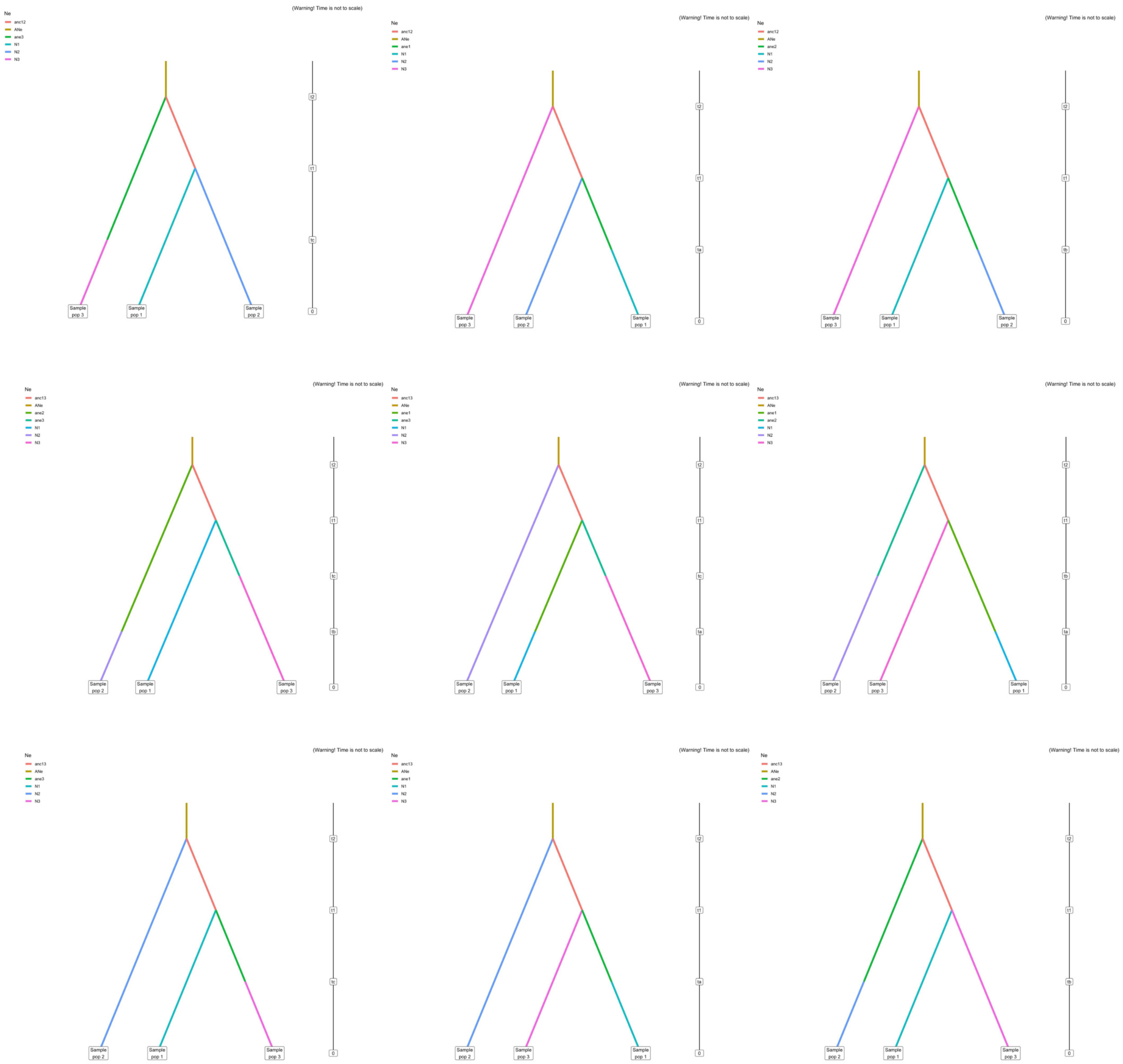


Figure S4 (Continued): Scenarios of Family 1 tested on the first step of ABC-RF to assess the early divergence and evolutionary relationships of Middle East, Caucasus, North African populations. Pop 1, pop2, and pop 3 correspond to Middle East, N. Africa and Caucasus, respectively.

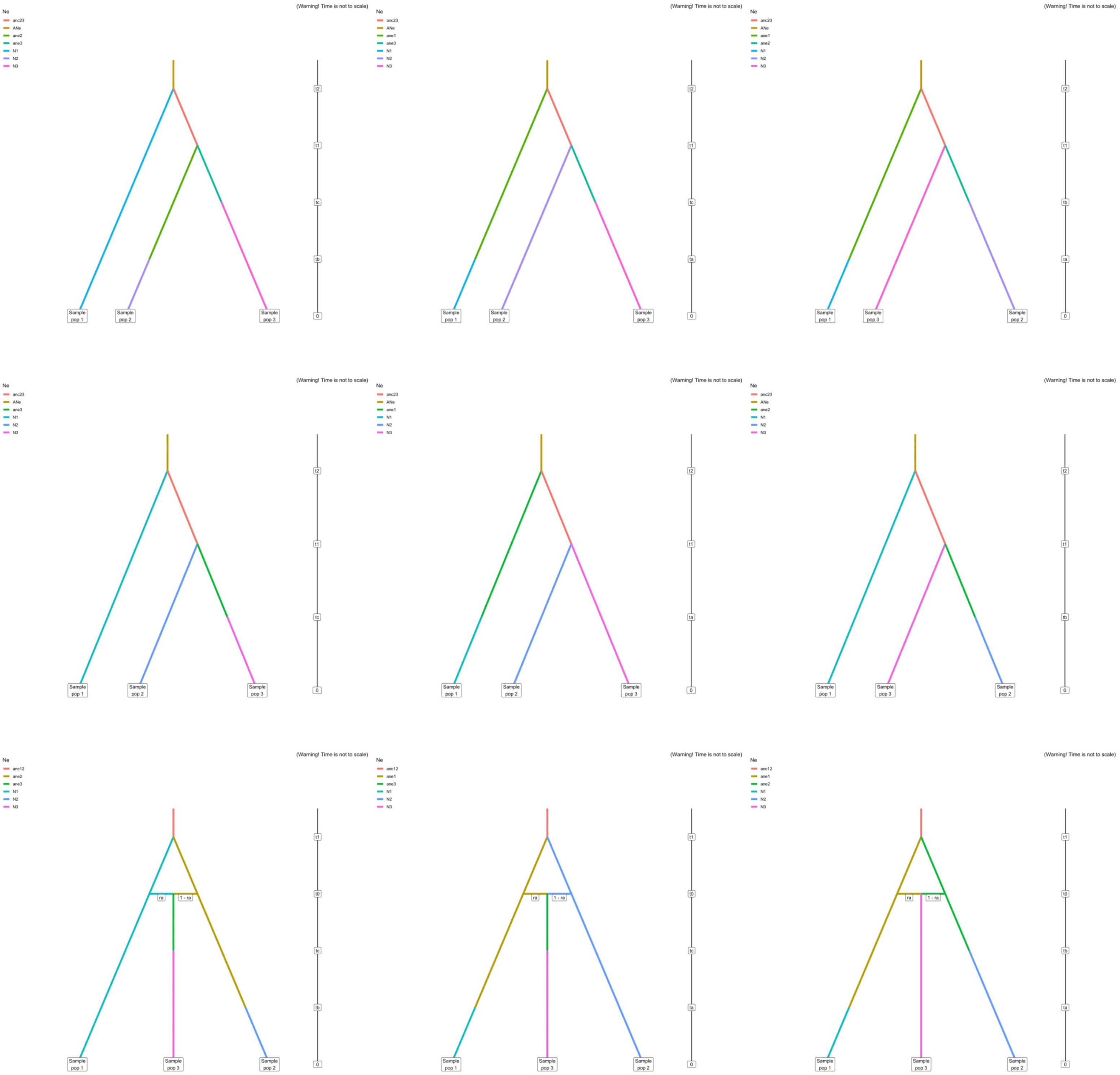


Figure S4 (Continued): Scenarios of Family 1 tested on the first step of ABC-RF to assess the early divergence and evolutionary relationships of Middle East, Caucasus, North African populations. Pop 1, pop2, and pop 3 correspond to Middle East, N. Africa and Caucasus, respectively.

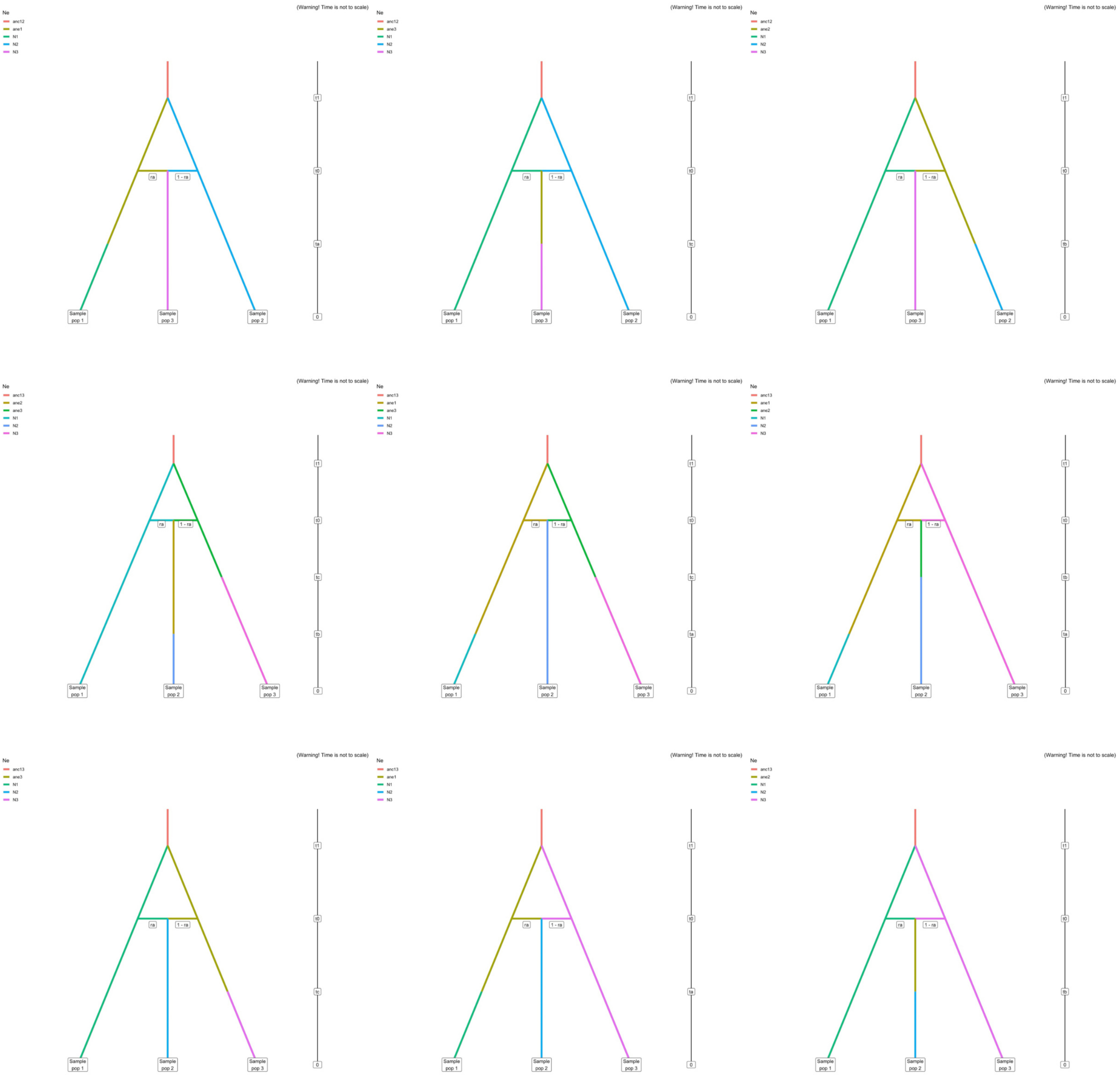


Figure S4 (Continued): Scenarios of Family 1 tested on the first step of ABC-RF to assess the early divergence and evolutionary relationships of Middle East, Caucasus, North African populations. Pop 1, pop2, and pop 3 correspond to Middle East, N. Africa and Caucasus, respectively.

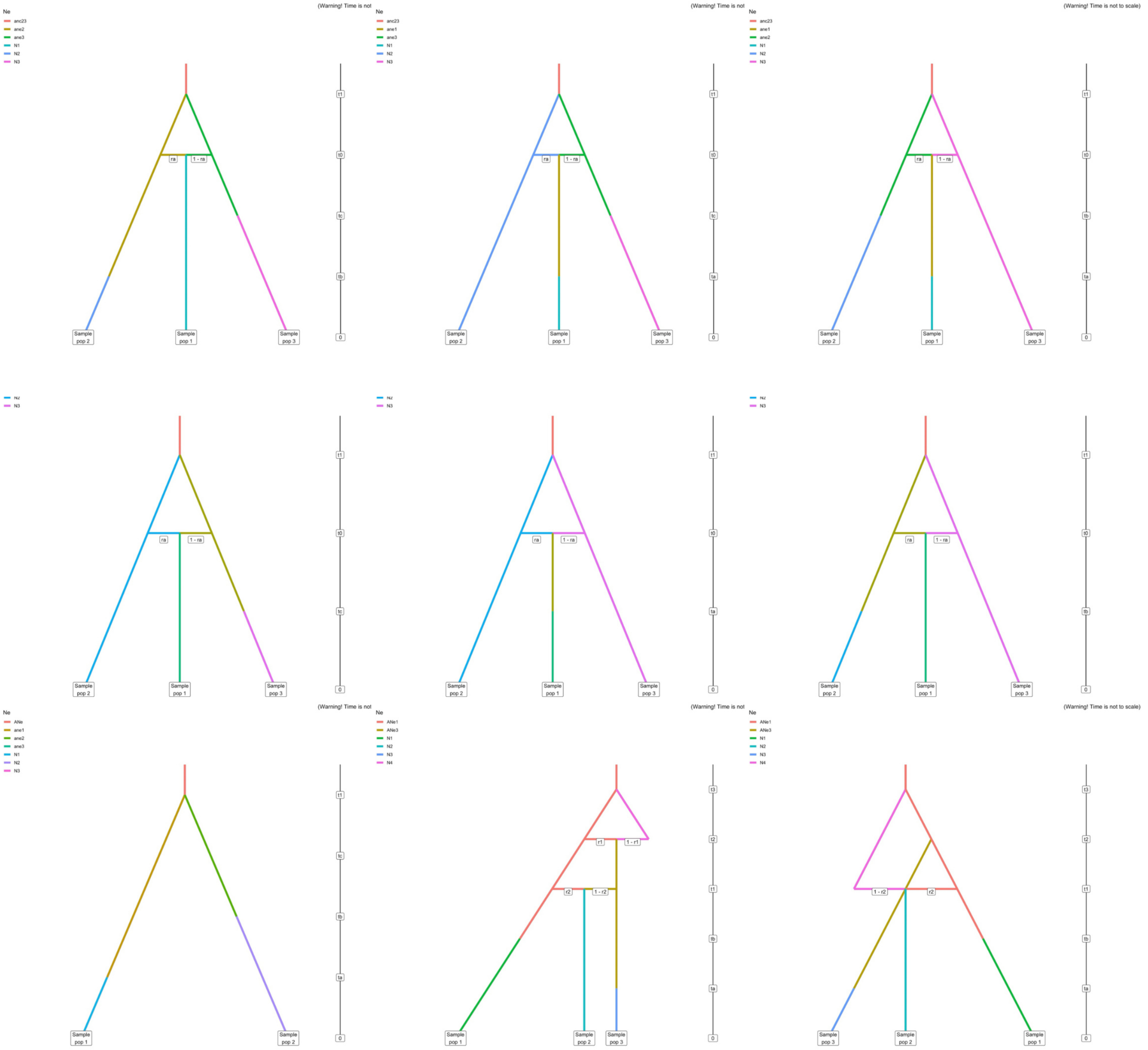

Figure S4 (Continued): Scenarios of Family 1 tested on the first step of ABC-RF to assess the early divergence and evolutionary relationships of Middle East, Caucasus, North African populations. Pop 1, pop2, and pop 3 correspond to Middle East, N. Africa and Caucasus, respectively.

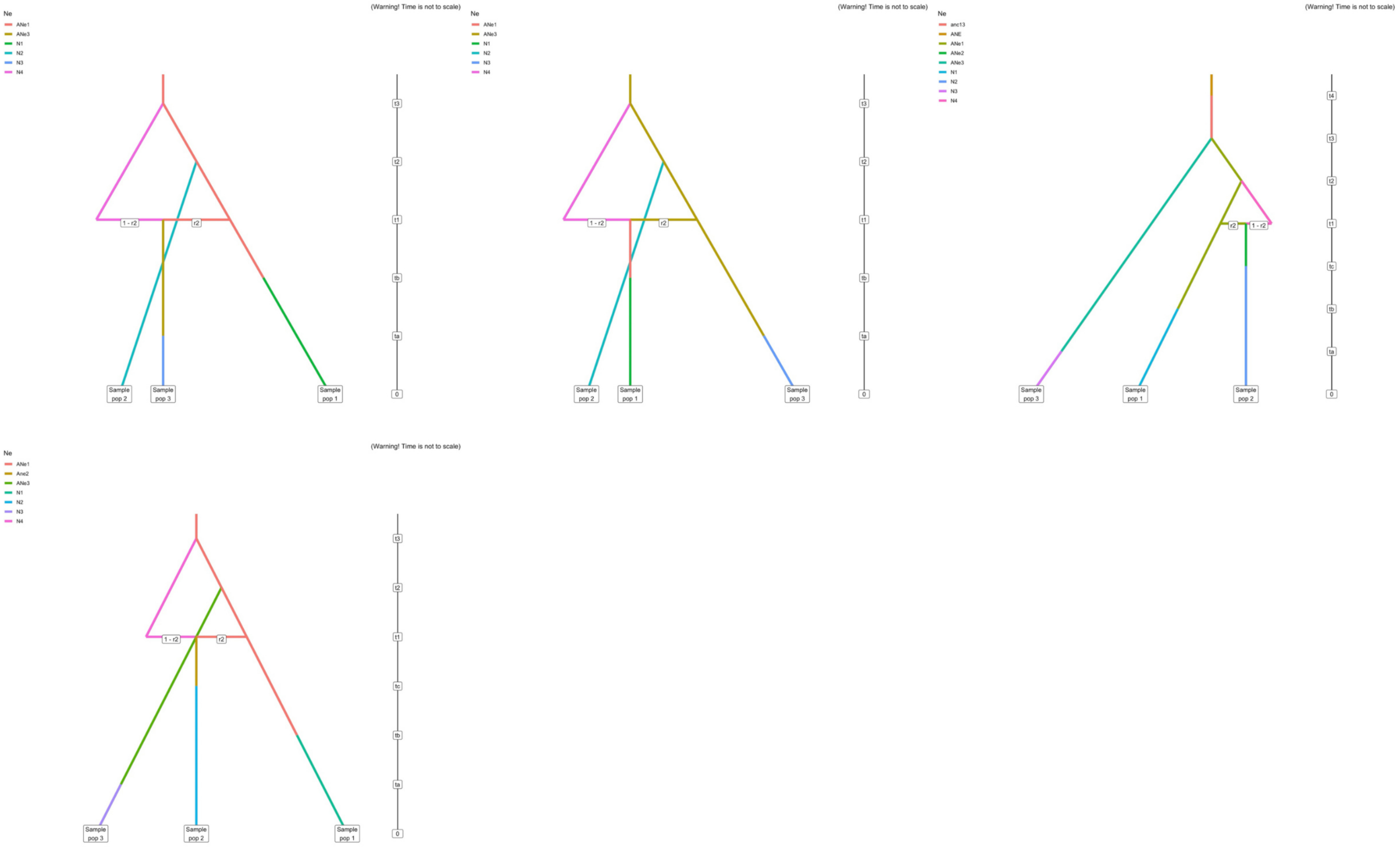


Figure S4 (Continued): Scenarios of Family 1 tested on the first step of ABC-RF to assess the early divergence and evolutionary relationships of Middle East, Caucasus, North African populations. Pop 1, pop2, and pop 3 correspond to Middle East, N. Africa and Caucasus, respectively.
