# Supplementary material for: Emergence and spread of the barley net blotch pathogen coincided with crop domestication and cultivation history
Source: PLoS Genet. 2024 Jan 29;20(1):e1010884. doi: 10.1371/journal.pgen.1010884 (PMC10852282; doi:10.1371/journal.pgen.1010884)
Supplement: S5 Fig — (PDF) [file pgen.1010884.s006.pdf]

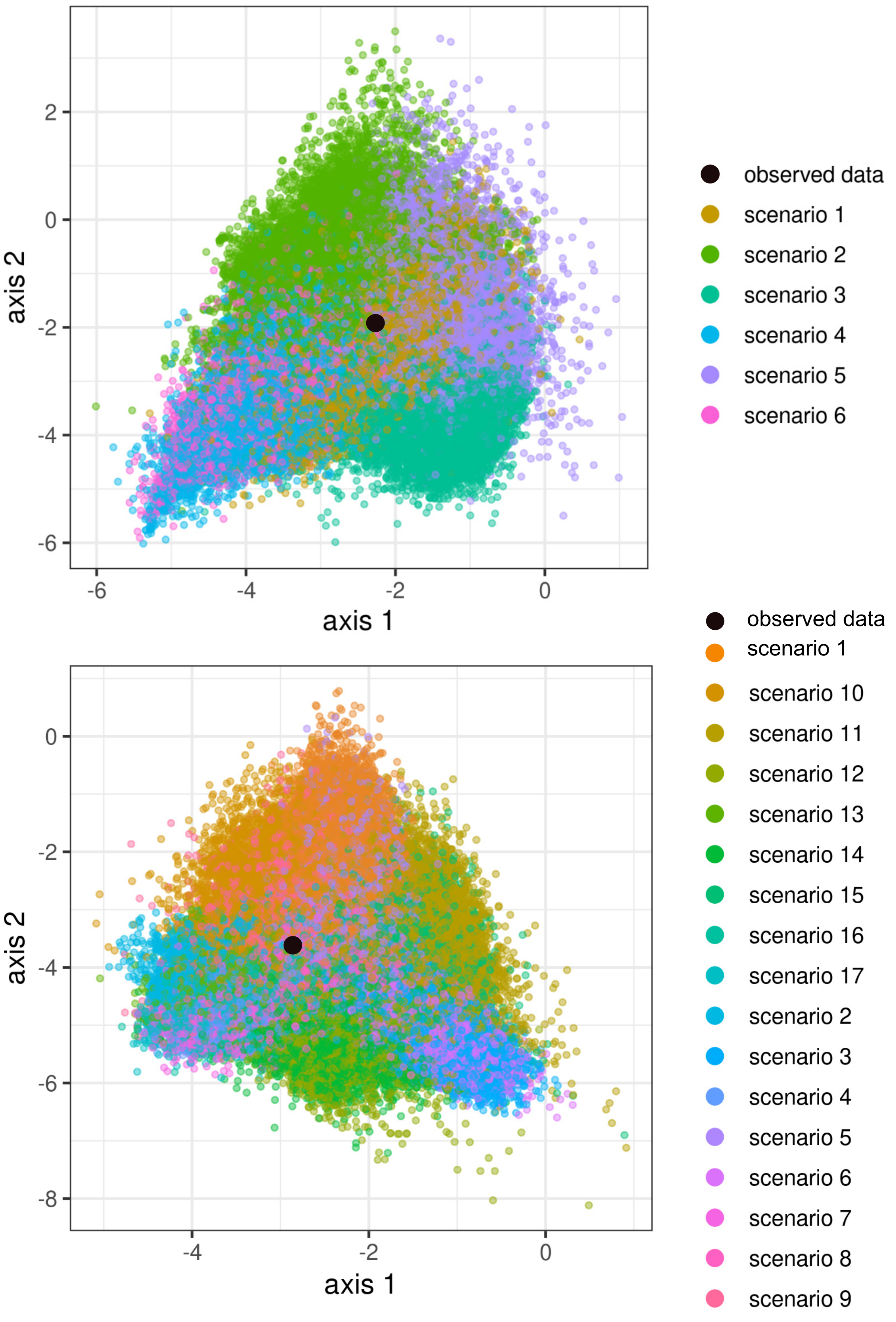

Figure S5: Scenarios of Family 2 tested on the second step of ABC-RF to assess the evolutionary relationship of the European with the *P. teres* f. *teres* populations. Pop 1, pop2, pop 3, and pop 4 correspond to Middle East, Europe, N. Africa, and Caucasus, respectively.

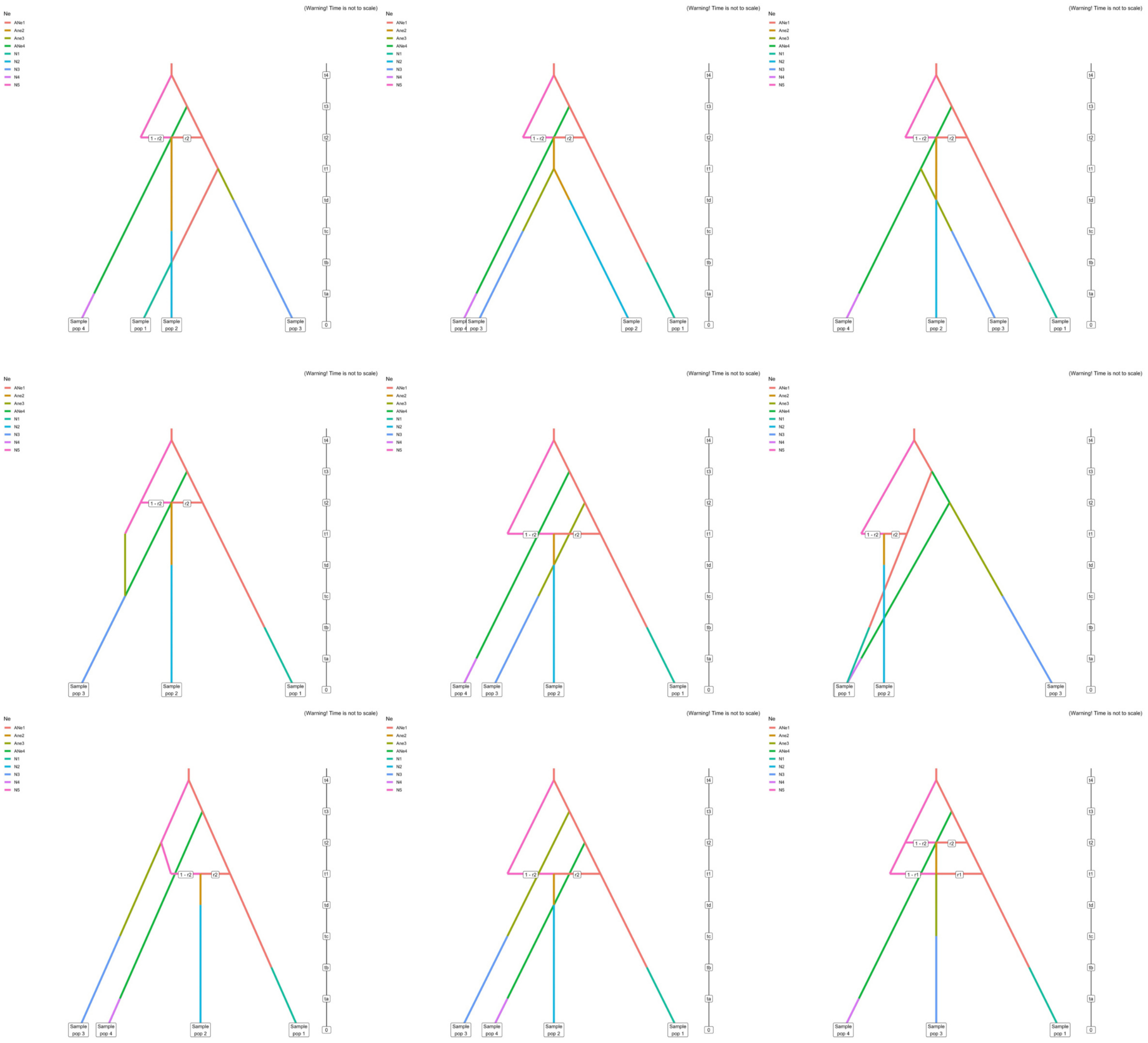

Figure S5 (Continued): Scenarios of Family 2 tested on the second step of ABC-RF to assess the evolutionary relationship of the European with the *P. teres* f. *teres* populations. Pop 1, pop2, pop 3, and pop 4 correspond to Middle East, Europe, N. Africa, and Caucasus, respectively.

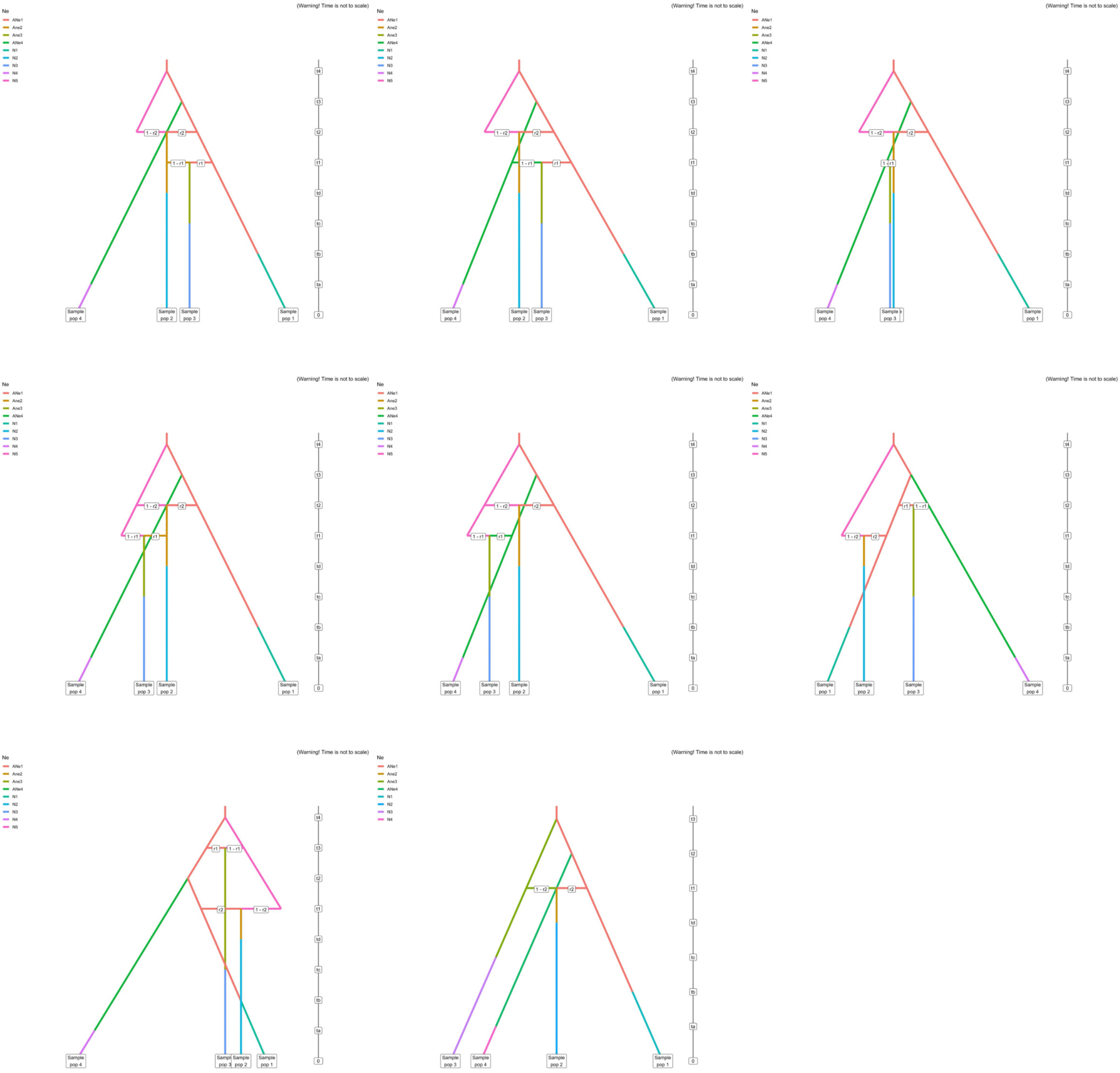

Figure S5 (Continued): Scenarios of Family 2 tested on the second step of ABC-RF to assess the evolutionary relationship of the European with the *P. teres* f. *teres* populations. Pop 1, pop2, pop 3, and pop 4 correspond to Middle East, Europe, N. Africa, and Caucasus, respectively.
