# Supplementary material for: Emergence and spread of the barley net blotch pathogen coincided with crop domestication and cultivation history
Source: PLoS Genet. 2024 Jan 29;20(1):e1010884. doi: 10.1371/journal.pgen.1010884 (PMC10852282; doi:10.1371/journal.pgen.1010884)
Supplement: S6 Fig — (PDF) [file pgen.1010884.s007.pdf]

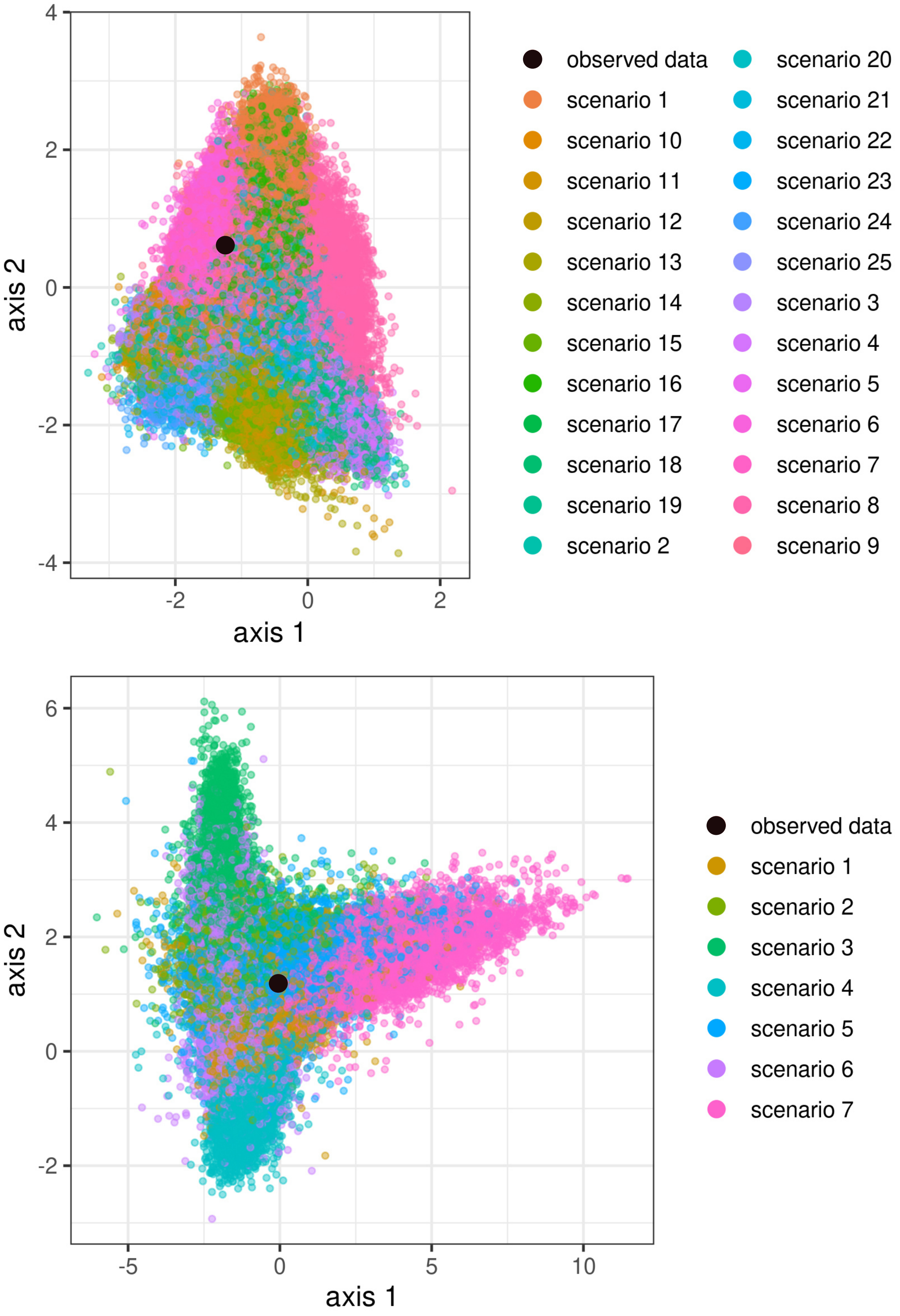

Figure S6: Scenarios of Family 3 tested on the third step of ABC-RF to assess the evolutionary relationship of the N. American with the *P. teres* f. *teres* populations. Pop 1, pop2, pop 3, pop 4, and pop 5 correspond to Middle East, Europe, N. Africa, N. America, and Caucasus, respectively.

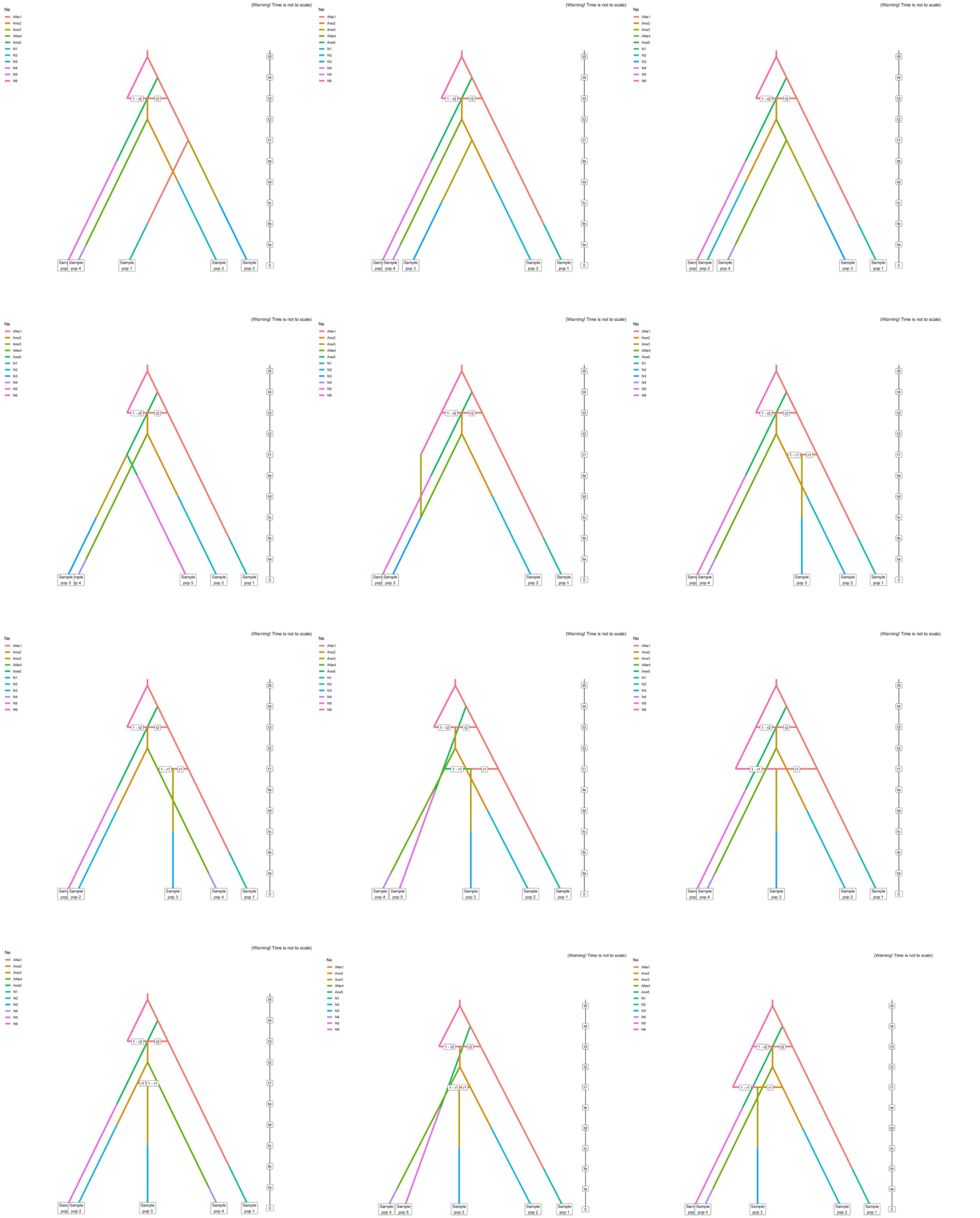

Figure S6 (Continued): Scenarios of Family 3 tested on the third step of ABC-RF to assess the evolutionary relationship of the N. American with the *P. teres* f. *teres* populations. Pop 1, pop2, pop 3, pop 4, and pop 5 correspond to Middle East, Europe, N. Africa, N. America, and Caucasus, respectively.

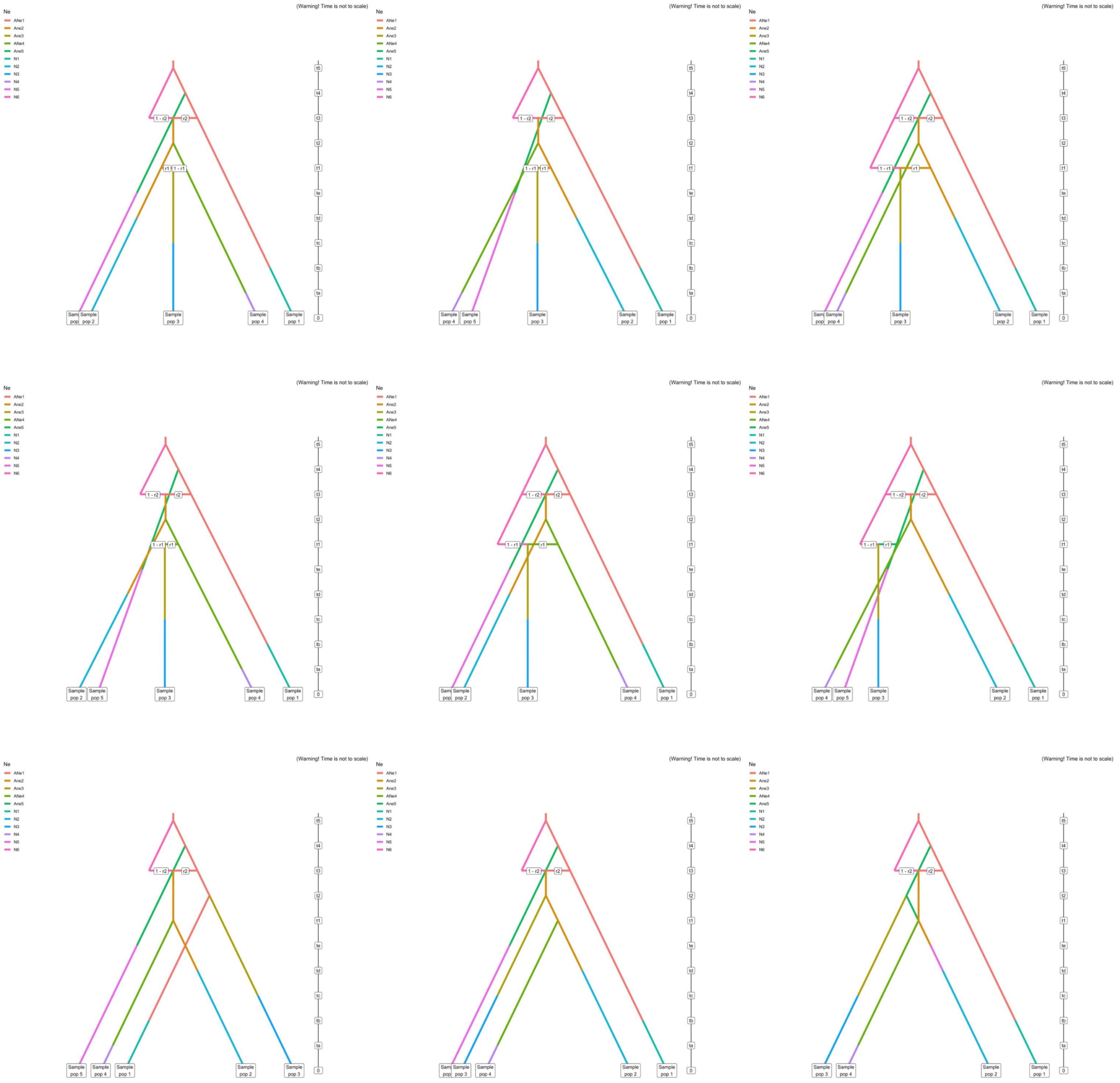

Figure S6 (Continued): Scenarios of Family 3 tested on the third step of ABC-RF to assess the evolutionary relationship of the N. American with the *P. teres* f. *teres* populations. Pop 1, pop2, pop 3, pop 4, and pop 5 correspond to Middle East, Europe, N. Africa, N. America, and Caucasus, respectively.

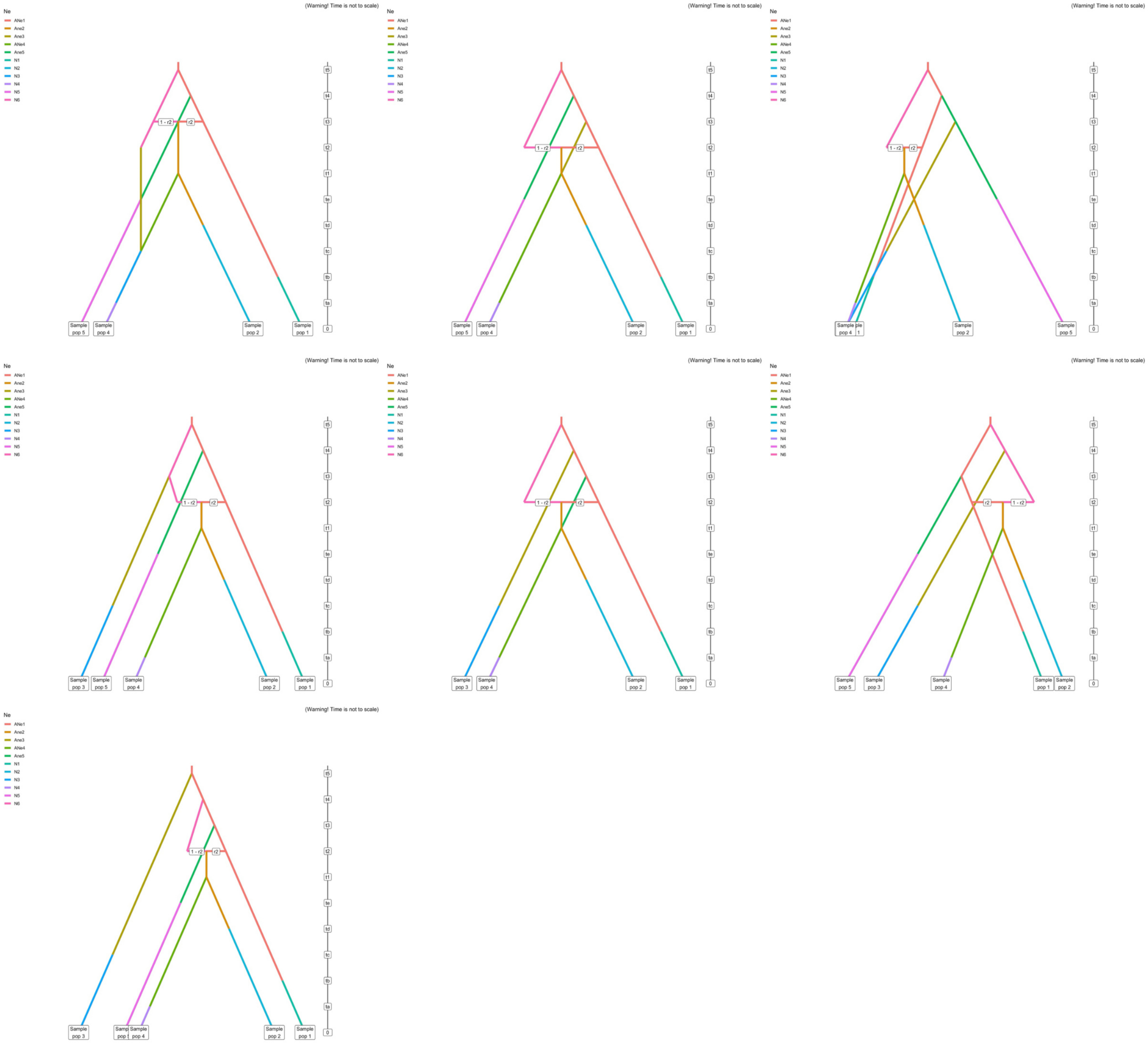

Figure S6 (Continued): Scenarios of Family 3 tested on the third step of ABC-RF to assess the evolutionary relationship of the N. American with the *P. teres* f. *teres* populations. Pop 1, pop2, pop 3, pop 4, and pop 5 correspond to Middle East, Europe, N. Africa, N. America, and Caucasus, respectively.
