# Supplementary material for: Emergence and spread of the barley net blotch pathogen coincided with crop domestication and cultivation history
Source: PLoS Genet. 2024 Jan 29;20(1):e1010884. doi: 10.1371/journal.pgen.1010884 (PMC10852282; doi:10.1371/journal.pgen.1010884)
Supplement: S7 Fig — (PDF) [file pgen.1010884.s008.pdf]

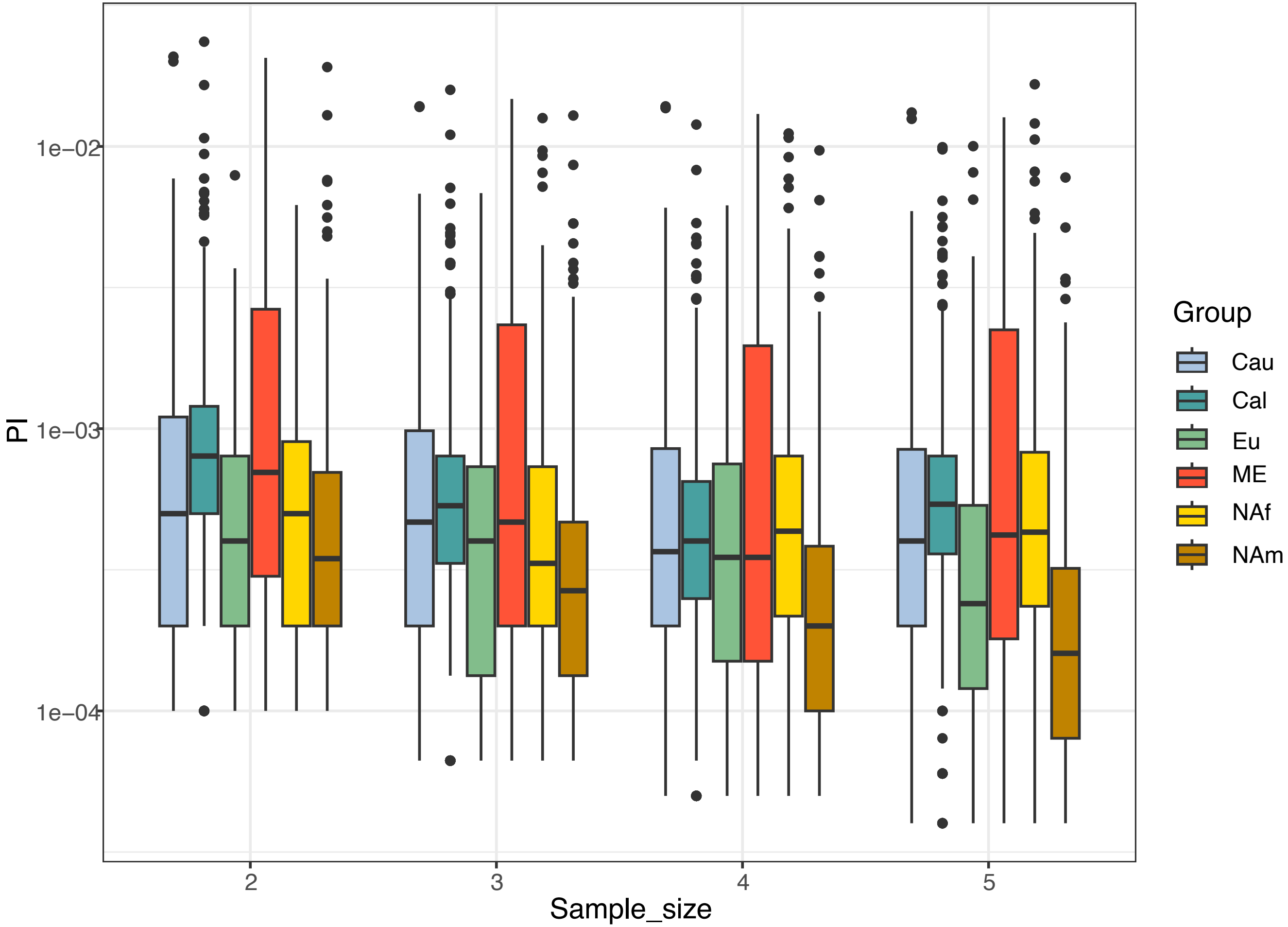

Figure S7: The effect of sample size on the genetic diversity estimations. to investigate the effect of sample size on the genetic diversity estimations, we followed a resample approach. We estimated genetic diversity in four independent rans, using 2, 3, 4, and 5 individuals per population.
