# Supplementary material for: Emergence and spread of the barley net blotch pathogen coincided with crop domestication and cultivation history
Source: PLoS Genet. 2024 Jan 29;20(1):e1010884. doi: 10.1371/journal.pgen.1010884 (PMC10852282; doi:10.1371/journal.pgen.1010884)
Supplement: Attachment — Submitted filename: Taliadoros_2ndReviewerResponse.pdf [file pgen.1010884.s010.pdf]

Reviewer #1: The authors have responded to most of my previous comments, but their new version of the manuscript raised some more questions:

Line 148: I would add mention to the range of coverage. Information on the minimum is important to know what was the sequencing quality of the worse sample included in the analyses

Reply: We thank the reviewer for this comment. Information about the minimum and maximum coverage in line 142.

Line 164: “significantly higher” need mention of parameters and results of a statistical test

Reply: We thank the reviewer for this comment. We added information about the statistical test and the p-values in line 168.

Lines 164-169: I find it weird to mention diversity levels for some populations but not for all

Reply: We thank the reviewer for pointing out that some values were missing from the text. The information was added in lines 168-174

Line 176: a Tajima’s D of 0.62 is hardly evidence for a bottleneck, especially in comparison to even higher Tajima’s D values in other populations

Reply: We thank the reviewer for this comment. We agree with this view and rephrased accordingly.

Line 178: this sentence has no background: “with a frequency of genetic variants that reflects a mutation – drift equilibrium ”. Assuming that this is based on Tajima’s D, I find the text very unclear without a direct mention to a Tajima’s D value and statistical test. The mention to table one is missing from this paragraph.

Reply: We thank the reviewer for this comment. We modified the text adding information about the Tajima’s D value and statistical test in lines 185-188

Lines 180-187: The authors messages are probably true, but I find the way they are presented weird. The North American population is discussed as having very long linkage blocks, and the value for r2/2 of 3.9 in Table 1 is mentioned as a reference. Except the population from the Caucasus has a much higher r2/2 of 7.3. When looking in more detail at figure 1c, this is likely due to a lower maximum r2 for Caucasus, which suggests that the measure reported is not the most relevant. I can see

in the figure what the authors mean, that the North American population has more LD, but the measures used to argue for this are unclear. Furthermore, long LD blocks for the North American population are interpreted as a recent founding of this population. However, Caucasus has much longer LD blocks (by the measure used by the authors), but is proposed to be a very ancient population.

Reply: We thank the reviewer for highlighting this issue. We have rewritten the text to clarify the relevance of LD and the differences among populations.

Line 195: How is random mating the null hypothesis here? The two mating types can only mate between each other, so that they should maintain a mating type ratio of 1:1. This is very far from random mating, which would not result in a 1:1 ratio

Reply: We have rephrased the sentence to underline the relevance of the mating type distribution in the fungal populations.

Line 199: typo at “in”

Reply: Corrected

Line 331: It is unclear what “these events” refer to. Since the authors are talking about population splits, I hardly see how “these events” coincide with domestication of barley, since they barely overlap. They seem to rather have followed domestication of barley, I.e. be more recent than domestication of barley, which is also a perfectly plausible biological scenario.

Reply: We thank the reviewer for this comment. We agree and rephrased now lines 352-353

Line 383: This sentence should be supported by the mention of a statistical test of significance

Reply: We thank the reviewer for this comment. We edited the line, adding information about the test (now line 400)

Lines 404-407: the simple overlap between QTL regions and selective sweeps demonstrates very little. Is the overlap larger than expected by chance?

Reply: We thank the reviewer for this comment. This might be beyond what we aimed to convey here. In the previous few lines, we wanted to highlight that almost half of the selective sweep regions which we identify in the weed-infecting population overlap with positions of selective sweeps in the crop-infecting populations. Since some of these regions have previously reported to be associated with virulence through a QTL analysis, we

speculate that the genes located in these genomic regions must be important for virulence on both wild and cultivated barley.
Further experimental studies would be necessary to validate this hypothesis.

Line 744: It is unclear what significance threshold the authors use for Simulations

Reply: We thank the reviewer for this comment. We added lines 766-767 to clarify this.
