# Supplementary material for: Emergence and spread of the barley net blotch pathogen coincided with crop domestication and cultivation history
Source: PLoS Genet. 2024 Jan 29;20(1):e1010884. doi: 10.1371/journal.pgen.1010884 (PMC10852282; doi:10.1371/journal.pgen.1010884)
Supplement: Attachment — Submitted filename: Taliadoros_ResponseToReviewers.docx [file pgen.1010884.s009.docx]

Reviewer #1: The Authors reconstruct the demographic history of the Barley net blotch pathogen using genomic data collected across continents, and they find signatures of selection at genomic regions enriched for effector genes likely involved in host-pathogen interactions.
I find the manuscript well written, I find the amount of work considerable and the analyses generally robust and that the results align with the authors claims, which are interesting and novel.
However, some parts were for me hard to follow, for example the discussion on the different models tested with ABC, and some results did not really behave as I would have expected, like the msmc2-ccr analysis, or where not really consistent across the manuscript, like for Ne estimated from theta (table 1) and with msmc2 (Fig 4c).

More specific comments below:

Table 1: for all populations, theta pi is larger than theta W. As a consequence, Tajima’s D values should all be positive, but in the table they are not. Maybe the authors can clarify this.

Reply: We thank the reviewer for bringing this to our attention. The genetic diversity estimators were calculated using a different software (ANGSD) than the Tajima’s D (vcftools). Variation in the estimation of the statistics can occur due to the different implementations of the statistics in the different software. We have recalculated Tajima’s D using ANGSD and indeed the estimations are all positive. For consistency reasons, we replaced the Tajima’s D values on Table 1, adjusted the lines 182-191, and figure S1, accordingly.

Results:
Line 163: “indicating” a bottleneck seems too strong. While a positive tajima’s D value is consistent with a bottleneck, other scenarios may result in a positive Tajima’s D. I would edit to “suggesting” a bottleneck scenario

Reply: We thank the reviewer for this comment. We have edited the line accordingly.

The section on ABC inference of population splits was for me difficult to follow. I understand the authors tested many different scenarios, but I found myself wondering which these scenarios are, why they were chosen, and what was the rational behind inferring this history in three steps, and including the European and North American populations only at the second and third steps. I think it would help the reader to try to restructure this part and discuss which scenarios were tested and why

Reply: We are grateful for this comment. Lines 291-296 were added to clarify the points that the reviewer raised here. Also, we have provided a detailed description of the scenarios of the model choice process, in the Methods section lines 657-730, in table S7, and figures S4-S6.

Line 310: this sentence refers to fig 4D, on the cross-coalescence rate, but my impression from the text was that the authors are still talking about inference of effective population size (would be fig 4c). This sentence could be restructured to clarify when the authors are estimating Ne and when split times.

Reply: The sentence (now line 366) was restructured to clarify estimations of changes in effective population size and splitting time.

Effective population size for the Caucasus has an interesting spike at very ancient times. The authors also showed earlier in the manuscript that in the Caucasus there are two diverged genetic clusters (from the neighbour-net-tree). I was wondering what part may the two clusters have in potentially creating this spike. Population structure results in lower coalescence rates, hence high inferred Ne, but this effect is normally seen in more recent times rather than in more ancient times. However the two clusters in the Caucasus seem to be highly diverged. I wonder if the authors included these two clusters in msmc2, and if they tried running msmc2 with the main cluster only to see if the spike in Ne remains or disappears.

Reply: We thank the reviewer for this comment. Because of the small sample size of the second Caucasian cluster (only three isolates), we decided to exclude the cluster from the inference of the demographic history and selection. So, the cluster is not included in the MSMC2 analysis as well. However, we report that this cluster exists and it would be very interesting to study the role of this cluster further, however we consider that this would rely on a more extensive sampling in the area.

The authors infer with msmc2 a trend in Ne that almost never goes higher than Ne=1000 for the majority of these populations histories, rather stays mostly under that. How do the authors interpret this in comparison to their table 1, where Ne is rather always higher than 13000 and sometimes up to 60000? Are the same parameters used in both analyses (e.g. mutation rate, 2*Ne*mu versus 4*Ne*mu for theta, 1/2*Ne versus 1/Ne for coalescence rates in diploids versus haploids)?

Reply: We thank the reviewer for highlighting this. Indeed, there was an error in the calculations of the Ne on Table 1. We re-calculated Ne using the formula: Ne = θ estimator / 2μ, where θ estimator is π and Watterson’s θ and μ is 5,7 x 10e-7, as used in the MSMC2 analysis. Now estimations on Table 1 are in agreement with the MSMC2 inference with estimations based on Watterson’s θ being between 1000-2000 individuals and a bit higher (between 2000-5000) when π is used as a genetic diversity estimator.

We have updated table 1 accordingly.

The trend lines for the cross-coalescence rate analyses seem to level around CCR=0.5 going backwards in time, starting around 10kya into the ancient past. These lines are supposed to level at ccr=1 plus minus some noise, when the populations merge into the ancestral population. I suggest the authors check their parameterisation of CCR, for whether there may be a factor of 2 set differently compared to other studies? This will probably not affect the authors claims in the text, since they focus on the relative age of splits, but it will affect the absolute values for split times and the clarity of the results

Reply: We revisited and revised this analysis. It seems that the issue highlighted by the reviewer here stemmed out of the different subsets used to calculate the coalescent rate (CR) 1) within the populations (initially, all isolates were used for this estimation) and 2) between populations (five replicates of seven randomly selected haplotypes per population). The relative cross-coalescence rate (RCCR) is the coalescence rate between populations over the average coalescence within populations and the abovementioned inconsistency has affected the RCCR calculations. To correct for the effect of the number of haplotypes, we re-estimated RCCR using the same haplotypes to calculated the CR within (calculated based on the seven randomly selected isolated for each population) and between populations (calculated on the combinations of the fourteen haplotypes) for each replicate run. Following this method we arrived at very similar results as in our previous analysis, with the trend lines now leveling closer to CCR= 1.

Figure 4D is replaced by the new version of the analysis. Methods section, lines 788-792 were adjusted accordingly.

Selection analysis:
Line 368: I found myself here asking why the authors did not make a GO analysis before this point, because this section sounds like the permutation test was focused on effector genes. However the authors mention a GO analysis in methods, and this appears only after they discuss selection signatures at effector genes. This is to me a bit of an odd structure for this section of results, where I would have expected a GO analysis right after the selection scans, and only after that a discussion of candidate genes under selection, including the effector genes. It makes me wonder, does a GO analysis pick effector genes?

Reply: We thank the reviewer for this comment. Unfortunately, most of the predicted effector genes used in the effector enrichment analysis do not have a GO term. The GO term enrichment analysis is done only for the subset of genes that have a GO term assigned to them. We consider that the lack of GO terms affects the enrichment analysis and produces biased results. Therefore, we decided to exclude the analysis from the manuscript.

Methods:
Line 534-539: It was not clear to me what is the difference between point 2 and 6
Reply: We have now clarified this in line 584.

In the methods for msmc2, I would expect to see some information on how many genomes were used and in which combinations (2,4,8 haplotypes mode, and what configuration for CCR). Some of this is mentioned in results and not in methods, while I would think this belongs to methods

Reply: We thank the reviewer for this comment. The information was added in lines 788-792.

Discussion:
The authors propose a link between the Caucasus and North America. Is a similar link known for Barley? I was missing some discussion of the history of Barley introduction to North America and whether this aligns with the genomic data of the fungus

Reply: We have added lines 498-504 speculating about the reasons behind the link of the Caucasian and the N. American populations

Minor comments:

1) Line 153: typo, that -> than

Corrected

Line 189 has a typo at “In”

Corrected
