# Supplementary material for: Emergence and spread of the barley net blotch pathogen coincided with crop domestication and cultivation history
Source: PLoS Genet. 2024 Jan 29;20(1):e1010884. doi: 10.1371/journal.pgen.1010884 (PMC10852282; doi:10.1371/journal.pgen.1010884)
Supplement: Peer review history

# Peer review: Decision Letter 0

**DOI:** 10.1371/journal.pgen.1010884.r001

**Authors:** Gregory P. Copenhaver

7 Sep 2023

Dear Eva,

I apologize for the length of time it has taken us to return our decision to you.  We had secured two external peer-reviewers but despite several reminders from the journal, and from me personally, one of them is unresponsive.  The review we have is thoughtful constructive, so using that together with our own judgment we have decided to move forward with a single review.  Below please find the standard letter inviting a revised manuscript.

-cheers

GPC

Dear Dr Stukenbrock,

Thank you very much for submitting your Research Article entitled 'Emergence and spread of the barley net blotch pathogen coincided with crop domestication and cultivation history' to PLOS Genetics.

The manuscript was fully evaluated at the editorial level and by independent peer reviewers. The reviewers appreciated the attention to an important problem, but raised some substantial concerns about the current manuscript. Based on the reviews, we will not be able to accept this version of the manuscript, but we would be willing to review a much-revised version. We cannot, of course, promise publication at that time.

Should you decide to revise the manuscript for further consideration here, your revisions should address the specific points made by each reviewer. We will also require a detailed list of your responses to the review comments and a description of the changes you have made in the manuscript.

If you decide to revise the manuscript for further consideration at PLOS Genetics, please aim to resubmit within the next 60 days, unless it will take extra time to address the concerns of the reviewers, in which case we would appreciate an expected resubmission date by email to plosgenetics@plos.org.

If present, accompanying reviewer attachments are included with this email; please notify the journal office if any appear to be missing. They will also be available for download from the link below. You can use this link to log into the system when you are ready to submit a revised version, having first consulted our Submission Checklist.

To enhance the reproducibility of your results, we recommend that you deposit your laboratory protocols in protocols.io, where a protocol can be assigned its own identifier (DOI) such that it can be cited independently in the future. Additionally, PLOS ONE offers an option to publish peer-reviewed clinical study protocols. Read more information on sharing protocols at https://plos.org/protocols?utm_medium=editorial-email&utm_source=authorletters&utm_campaign=protocols

Please be aware that our data availability policy requires that all numerical data underlying graphs or summary statistics are included with the submission, and you will need to provide this upon resubmission if not already present. In addition, we do not permit the inclusion of phrases such as "data not shown" or "unpublished results" in manuscripts. All points should be backed up by data provided with the submission.

While revising your submission, please upload your figure files to the Preflight Analysis and Conversion Engine (PACE) digital diagnostic tool.  PACE helps ensure that figures meet PLOS requirements. To use PACE, you must first register as a user. Then, login and navigate to the UPLOAD tab, where you will find detailed instructions on how to use the tool. If you encounter any issues or have any questions when using PACE, please email us at figures@plos.org.

PLOS has incorporated Similarity Check, powered by iThenticate, into its journal-wide submission system in order to screen submitted content for originality before publication. Each PLOS journal undertakes screening on a proportion of submitted articles. You will be contacted if needed following the screening process.

To resubmit, use the link below and 'Revise Submission' in the 'Submissions Needing Revision' folder.

We are sorry that we cannot be more positive about your manuscript at this stage. Please do not hesitate to contact us if you have any concerns or questions.

Yours sincerely,

Gregory P. Copenhaver

Editor-in-Chief

PLOS Genetics

Gregory Barsh

Editor-in-Chief

PLOS Genetics

Reviewer's Responses to Questions

**Comments to the Authors:**

**Please note here if the review is uploaded as an attachment.**

Reviewer #1: The Authors reconstruct the demographic history of the Barley net blotch pathogen using genomic data collected across continents, and they find signatures of selection at genomic regions enriched for effector genes likely involved in host-pathogen interactions.

I find the manuscript well written, I find the amount of work considerable and the analyses generally robust and that the results align with the authors claims, which are interesting and novel.

However, some parts were for me hard to follow, for example the discussion on the different models tested with ABC, and some results did not really behave as I would have expected, like the msmc2-ccr analysis, or where not really consistent across the manuscript, like for Ne estimated from theta (table 1) and with msmc2 (Fig 4c).

More specific comments below:

Table 1: for all populations, theta pi is larger than theta W. As a consequence, Tajima’s D values should all be positive, but in the table they are not. Maybe the authors can clarify this.

Results:

Line 163: “indicating” a bottleneck seems too strong. While a positive tajima’s D value is consistent with a bottleneck, other scenarios may result in a positive Tajima’s D. I would edit to “suggesting” a bottleneck scenario

The section on ABC inference of population splits was for me difficult to follow. I understand the authors tested many different scenarios, but I found myself wondering which these scenarios are, why they were chosen, and what was the rational behind inferring this history in three steps, and including the European and North American populations only at the second and third steps. I think it would help the reader to try to restructure this part and discuss which scenarios were tested and why

Line 310: this sentence refers to fig 4D, on the cross-coalescence rate, but my impression from the text was that the authors are still talking about inference of effective population size (would be fig 4c). This sentence could be restructured to clarify when the authors are estimating Ne and when split times.

Effective population size for the Caucasus has an interesting spike at very ancient times. The authors also showed earlier in the manuscript that in the Caucasus there are two diverged genetic clusters (from the neighbour-net-tree). I was wondering what part may the two clusters have in potentially creating this spike. Population structure results in lower coalescence rates, hence high inferred Ne, but this effect is normally seen in more recent times rather than in more ancient times. However the two clusters in the Caucasus seem to be highly diverged. I wonder if the authors included these two clusters in msmc2, and if they tried running msmc2 with the main cluster only to see if the spike in Ne remains or disappears.

The authors infer with msmc2 a trend in Ne that almost never goes higher than Ne=1000 for the majority of these populations histories, rather stays mostly under that. How do the authors interpret this in comparison to their table 1, where Ne is rather always higher than 13000 and sometimes up to 60000? Are the same parameters used in both analyses (e.g. mutation rate, 2*Ne*mu versus 4*Ne*mu for theta, 1/2*Ne versus 1/Ne for coalescence rates in diploids versus haploids)?

The trend lines for the cross-coalescence rate analyses seem to level around CCR=0.5 going backwards in time, starting around 10kya into the ancient past. These lines are supposed to level at ccr=1 plus minus some noise, when the populations merge into the ancestral population. I suggest the authors check their parameterisation of CCR, for whether there may be a factor of 2 set differently compared to other studies? This will probably not affect the authors claims in the text, since they focus on the relative age of splits, but it will affect the absolute values for split times and the clarity of the results

Selection analysis:

Line 368: I found myself here asking why the authors did not make a GO analysis before this point, because this section sounds like the permutation test was focused on effector genes. However the authors mention a GO analysis in methods, and this appears only after they discuss selection signatures at effector genes. This is to me a bit of an odd structure for this section of results, where I would have expected a GO analysis right after the selection scans, and only after that a discussion of candidate genes under selection, including the effector genes. It makes me wonder, does a GO analysis pick effector genes?

Methods:

Line 534-539: It was not clear to me what is the difference between point 2 and 6

In the methods for msmc2, I would expect to see some information on how many genomes were used and in which combinations (2,4,8 haplotypes mode, and what configuration for CCR). Some of this is mentioned in results and not in methods, while I would think this belongs to methods

Discussion:

The authors propose a link between the Caucasus and North America. Is a similar link known for Barley? I was missing some discussion of the history of Barley introduction to North America and whether this aligns with the genomic data of the fungus

Minor comments:

1) Line 153: typo, that -> than

Line 189 has a typo at “In”

**********

**Have all data underlying the figures and results presented in the manuscript been provided?**

Large-scale datasets should be made available via a public repository as described in the *PLOS Genetics* data availability policy, and numerical data that underlies graphs or summary statistics should be provided in spreadsheet form as supporting information.

Reviewer #1: None

**********

PLOS authors have the option to publish the peer review history of their article (what does this mean?). If published, this will include your full peer review and any attached files.

If you choose “no”, your identity will remain anonymous but your review may still be made public.

**Do you want your identity to be public for this peer review?** For information about this choice, including consent withdrawal, please see our Privacy Policy.

Reviewer #1: No

# Author comment: Author response to Decision Letter 0

**DOI:** 10.1371/journal.pgen.1010884.r002

18 Oct 2023

Attachment Submitted filename: Taliadoros_ResponseToReviewers.docx Click here for additional data file.

# Peer review: Decision Letter 1

**DOI:** 10.1371/journal.pgen.1010884.r003

**Authors:** Gregory P. Copenhaver

7 Nov 2023

Dear Eva,

Thank you very much for submitting your Research Article entitled 'Emergence and spread of the barley net blotch pathogen coincided with crop domestication and cultivation history' to PLOS Genetics.

The manuscript was fully evaluated at the editorial level and by the original independent peer reviewer. The reviewer appreciated your revisions but identified some concerns that we ask you address in a revised manuscript. I think most of the reviewer comments can be handled by carefully and thoughtfully amending the text, but some re-analysis of data and supporting statistical analysis may also be necessary.

We therefore ask you to modify the manuscript according to the review recommendations. Your revisions should address the specific points made by each reviewer.

In addition we ask that you:

1) Provide a detailed list of your responses to the review comments and a description of the changes you have made in the manuscript.

2) Upload a Striking Image with a corresponding caption to accompany your manuscript if one is available (either a new image or an existing one from within your manuscript). If this image is judged to be suitable, it may be featured on our website. Images should ideally be high resolution, eye-catching, single panel square images. For examples, please browse our archive. If your image is from someone other than yourself, please ensure that the artist has read and agreed to the terms and conditions of the Creative Commons Attribution License. Note: we cannot publish copyrighted images.

We hope to receive your revised manuscript within the next 30 days. If you anticipate any delay in its return, we would ask you to let us know the expected resubmission date by email to plosgenetics@plos.org.

If present, accompanying reviewer attachments should be included with this email; please notify the journal office if any appear to be missing. They will also be available for download from the link below. You can use this link to log into the system when you are ready to submit a revised version, having first consulted our Submission Checklist.

While revising your submission, please upload your figure files to the Preflight Analysis and Conversion Engine (PACE) digital diagnostic tool. PACE helps ensure that figures meet PLOS requirements. To use PACE, you must first register as a user. Then, login and navigate to the UPLOAD tab, where you will find detailed instructions on how to use the tool. If you encounter any issues or have any questions when using PACE, please email us at figures@plos.org.

Please be aware that our data availability policy requires that all numerical data underlying graphs or summary statistics are included with the submission, and you will need to provide this upon resubmission if not already present. In addition, we do not permit the inclusion of phrases such as "data not shown" or "unpublished results" in manuscripts. All points should be backed up by data provided with the submission.

To enhance the reproducibility of your results, we recommend that you deposit your laboratory protocols in protocols.io, where a protocol can be assigned its own identifier (DOI) such that it can be cited independently in the future. Additionally, PLOS ONE offers an option to publish peer-reviewed clinical study protocols. Read more information on sharing protocols at https://plos.org/protocols?utm_medium=editorial-email&utm_source=authorletters&utm_campaign=protocols

Please review your reference list to ensure that it is complete and correct. If you have cited papers that have been retracted, please include the rationale for doing so in the manuscript text, or remove these references and replace them with relevant current references. Any changes to the reference list should be mentioned in the rebuttal letter that accompanies your revised manuscript. If you need to cite a retracted article, indicate the article’s retracted status in the References list and also include a citation and full reference for the retraction notice.

PLOS has incorporated Similarity Check, powered by iThenticate, into its journal-wide submission system in order to screen submitted content for originality before publication. Each PLOS journal undertakes screening on a proportion of submitted articles. You will be contacted if needed following the screening process.

To resubmit, you will need to go to the link below and 'Revise Submission' in the 'Submissions Needing Revision' folder.

Please let us know if you have any questions while making these revisions.

Yours sincerely,

Gregory P. Copenhaver

Editor-in-Chief

PLOS Genetics

Gregory Barsh

Editor-in-Chief

PLOS Genetics

Reviewer's Responses to Questions

**Comments to the Authors:**

**Please note here if the review is uploaded as an attachment.**

Reviewer #1: The authors have responded to most of my previous comments, but their new version of the manuscript raised some more questions:

Line 148: I would add mention to the range of coverage. Information on the minimum is important to know what was the sequencing quality of the worse sample included in the analyses

Line 164: “significantly higher” need mention of parameters and results of a statistical test

Lines 164-169: I find it weird to mention diversity levels for some populations but not for all

Line 176: a Tajima’s D of 0.62 is hardly evidence for a bottleneck, especially in comparison to even higher Tajima’s D values in other populations

Line 178: this sentence has no background: “with a frequency of genetic variants that reflects a mutation – drift equilibrium ”. Assuming that this is based on Tajima’s D, I find the text very unclear without a direct mention to a Tajima’s D value and statistical test. The mention to table one is missing from this paragraph.

Lines 180-187: The authors messages are probably true, but I find the way they are presented weird. The North American population is discussed as having very long linkage blocks, and the value for r2/2 of 3.9 in Table 1 is mentioned as a reference. Except the population from the Caucasus has a much higher r2/2 of 7.3. When looking in more detail at figure 1c, this is likely due to a lower maximum r2 for Caucasus, which suggests that the measure reported is not the most relevant. I can see in the figure what the authors mean, that the North American population has more LD, but the measures used to argue for this are unclear. Furthermore, long LD blocks for the North American population are interpreted as a recent founding of this population. However, Caucasus has much longer LD blocks (by the measure used by the authors), but is proposed to be a very ancient population.

Line 195: How is random mating the null hypothesis here? The two mating types can only mate between each other, so that they should maintain a mating type ratio of 1:1. This is very far from random mating, which would not result in a 1:1 ratio

Line 199: typo at “in”

Line 331: It is unclear what “these events” refer to. Since the authors are talking about population splits, I hardly see how “these events” coincide with domestication of barley, since they barely overlap. They seem to rather have followed domestication of barley, I.e. be more recent than domestication of barley, which is also a perfectly plausible biological scenario.

Line 383: This sentence should be supported by the mention of a statistical test of significance

Lines 404-407: the simple overlap between QTL regions and selective sweeps demonstrates very little. Is the overlap larger than expected by chance?

Line 744: It is unclear what significance threshold the authors use for simulations

**********

**Have all data underlying the figures and results presented in the manuscript been provided?**

Large-scale datasets should be made available via a public repository as described in the *PLOS Genetics* data availability policy, and numerical data that underlies graphs or summary statistics should be provided in spreadsheet form as supporting information.

Reviewer #1: Yes

**********

PLOS authors have the option to publish the peer review history of their article (what does this mean?). If published, this will include your full peer review and any attached files.

If you choose “no”, your identity will remain anonymous but your review may still be made public.

**Do you want your identity to be public for this peer review?** For information about this choice, including consent withdrawal, please see our Privacy Policy.

Reviewer #1: No

# Author comment: Author response to Decision Letter 1

**DOI:** 10.1371/journal.pgen.1010884.r004

7 Dec 2023

Attachment Submitted filename: Taliadoros_2ndReviewerResponse.pdf Click here for additional data file.

# Editor report: Decision Letter 2

**DOI:** 10.1371/journal.pgen.1010884.r005

**Authors:** Gregory P. Copenhaver

11 Dec 2023

Dear Dr Stukenbrock,

We are pleased to inform you that your manuscript entitled "Emergence and spread of the barley net blotch pathogen coincided with crop domestication and cultivation history" has been editorially accepted for publication in PLOS Genetics. Congratulations!

Before your submission can be formally accepted and sent to production you will need to complete our formatting changes, which you will receive in a follow up email. Please be aware that it may take several days for you to receive this email; during this time no action is required by you. Please note: the accept date on your published article will reflect the date of this provisional acceptance, but your manuscript will not be scheduled for publication until the required changes have been made.

Once your paper is formally accepted, an uncorrected proof of your manuscript will be published online ahead of the final version, unless you’ve already opted out via the online submission form. If, for any reason, you do not want an earlier version of your manuscript published online or are unsure if you have already indicated as such, please let the journal staff know immediately at plosgenetics@plos.org.

In the meantime, please log into Editorial Manager at https://www.editorialmanager.com/pgenetics/, click the "Update My Information" link at the top of the page, and update your user information to ensure an efficient production and billing process. Note that PLOS requires an ORCID iD for all corresponding authors. Therefore, please ensure that you have an ORCID iD and that it is validated in Editorial Manager. To do this, go to ‘Update my Information’ (in the upper left-hand corner of the main menu), and click on the Fetch/Validate link next to the ORCID field.  This will take you to the ORCID site and allow you to create a new iD or authenticate a pre-existing iD in Editorial Manager.

If you have a press-related query, or would like to know about making your underlying data available (as you will be aware, this is required for publication), please see the end of this email. If your institution or institutions have a press office, please notify them about your upcoming article at this point, to enable them to help maximise its impact. Inform journal staff as soon as possible if you are preparing a press release for your article and need a publication date.

Thank you again for supporting open-access publishing; we are looking forward to publishing your work in PLOS Genetics!

Yours sincerely,

Gregory P. Copenhaver

Editor-in-Chief

PLOS Genetics

Gregory Barsh

Editor-in-Chief

PLOS Genetics

www.plosgenetics.org

Twitter: @PLOSGenetics

----------------------------------------------------

Comments from the reviewers (if applicable):

----------------------------------------------------

**Data Deposition**

If you have submitted a Research Article or Front Matter that has associated data that are not suitable for deposition in a subject-specific public repository (such as GenBank or ArrayExpress), one way to make that data available is to deposit it in the Dryad Digital Repository. As you may recall, we ask all authors to agree to make data available; this is one way to achieve that. A full list of recommended repositories can be found on our website.

The following link will take you to the Dryad record for your article, so you won't have to re‐enter its bibliographic information, and can upload your files directly: 

http://datadryad.org/submit?journalID=pgenetics&manu=PGENETICS-D-23-00842R2

More information about depositing data in Dryad is available at http://www.datadryad.org/depositing. If you experience any difficulties in submitting your data, please contact help@datadryad.org for support.

Additionally, please be aware that our data availability policy requires that all numerical data underlying display items are included with the submission, and you will need to provide this before we can formally accept your manuscript, if not already present.

----------------------------------------------------

**Press Queries**

If you or your institution will be preparing press materials for this manuscript, or if you need to know your paper's publication date for media purposes, please inform the journal staff as soon as possible so that your submission can be scheduled accordingly. Your manuscript will remain under a strict press embargo until the publication date and time. This means an early version of your manuscript will not be published ahead of your final version. PLOS Genetics may also choose to issue a press release for your article. If there's anything the journal should know or you'd like more information, please get in touch via plosgenetics@plos.org.

# Editor report: Acceptance letter

**DOI:** 10.1371/journal.pgen.1010884.r006

**Authors:** Gregory P. Copenhaver

16 Jan 2024

PGENETICS-D-23-00842R2 

Emergence and spread of the barley net blotch pathogen coincided with crop domestication and cultivation history 

Dear Dr Stukenbrock, 

We are pleased to inform you that your manuscript entitled "Emergence and spread of the barley net blotch pathogen coincided with crop domestication and cultivation history" has been formally accepted for publication in PLOS Genetics! Your manuscript is now with our production department and you will be notified of the publication date in due course.

The corresponding author will soon be receiving a typeset proof for review, to ensure errors have not been introduced during production. Please review the PDF proof of your manuscript carefully, as this is the last chance to correct any errors. Please note that major changes, or those which affect the scientific understanding of the work, will likely cause delays to the publication date of your manuscript. 

Soon after your final files are uploaded, unless you have opted out or your manuscript is a front-matter piece, the early version of your manuscript will be published online. The date of the early version will be your article's publication date. The final article will be published to the same URL, and all versions of the paper will be accessible to readers.

Thank you again for supporting PLOS Genetics and open-access publishing. We are looking forward to publishing your work! 

With kind regards,

Zsofi Zombor

PLOS Genetics

On behalf of:

The PLOS Genetics Team

Carlyle House, Carlyle Road, Cambridge CB4 3DN | United Kingdom

plosgenetics@plos.org | +44 (0) 1223-442823

plosgenetics.org | Twitter: @PLOSGenetics
